# A novel empeROR of pancreatic malignancy

**DOI:** 10.15252/embj.2023114282

**Published:** 2023-06-14

**Authors:** Irene Peñuelas‐Haro, Elisa Espinet

**Affiliations:** ^1^ Department of Pathology and Experimental Therapy, School of Medicine University of Barcelona (UB) Barcelona Spain; ^2^ Molecular Mechanisms and Experimental Therapy in Oncology Program (Oncobell) Institut d'Investigació Biomèdica de Bellvitge (IDIBELL) Barcelona Spain

**Keywords:** Cancer

## Abstract

How pancreatic cancer cells acquire tumor initiating capacities remains poorly understood. A recent study by Yamazaki *et al* (2023) uncovers a crucial, targetable role of tyrosine kinase‐like orphan receptor (ROR1) in PDAC tumor formation and progression.

## Graphical abstract

Recent work reports tyrosine kinase receptor ROR1 as a functional marker of aggressive partial EMT‐type cancer cells in PDAC, boosting proliferation in a c‐Myc/E2F/AURKB‐dependent manner.

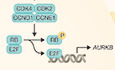

Pancreatic ductal adenocarcinoma (PDAC) is the most common form of pancreatic cancer, and it represents one of the most aggressive tumors nowadays. Despite recent advances in cancer therapy, therapeutic options for PDAC patients remain limited and the 5‐year survival rate is still below 10%. A proper understanding of the molecular signatures and the identification of altered signaling pathways in PDAC is crucial for the development of targeted therapies to effectively treat the disease. Large‐scale molecular profiling approaches have enabled the identification of multiple genomic, epigenomic, and transcriptomic alterations in PDAC tumors and derived metastases (Connor & Gallinger, 2022). These studies have started to reveal an unprecedented level of inter‐ and intratumor heterogeneity. Unfortunately, with counted exceptions, the increasing recognition of PDAC's complexities has not yet translated into successful personalized therapeutic strategies (Hosein *et al*, 2022). Accumulated evidence suggests that PDAC aggressiveness relates to the basal‐like transcriptomic subtype, and it is at least partly related to the activation of the epithelial‐to‐mesenchymal transition (EMT) phenotype (Chan‐Seng‐Yue *et al*, 2020). Basal‐like tumors are typically poorly differentiated, highly metastatic, and resistant to therapy (Collisson *et al*, 2019). Notably, EMT appears to be a major driver of cellular plasticity and intratumoral heterogeneity as reflected by multiple intermediate phenotypic EMT states coexisting simultaneously within a tumor (Pastushenko *et al*, 2021). The dynamic changes among different EMT states suggest a crucial role of this process to the appearance of cancer cells with stem‐like features. In fact, several studies have shown that PDAC heterogeneity is partly explained by a subset of cells named tumor initiating cells (TIC) or cancer stem cells (CSC). This subpopulation possesses highly plastic properties, such as self‐renewal and differentiation potential, which allow them to survive under adverse conditions and recapitulate histological hierarchy organization when needed. Consequently, TICs should be considered as plausible drug targets. Unfortunately, therapies proposed against TICs have so far not proven effective in the clinical setting, reinforcing the necessity to better understand which factors can mediate cellular plasticity.

A recent study by Yamazaki *et al* (2023) tackled this task by generating a xenograft model of the human S2‐VP10 PDAC cell line and performing extensive single‐cell RNA sequencing (scRNA‐seq) analyses. Expression profiling of the isolated neoplastic compartment revealed a cluster of tumor cells that presented a partial EMT signature. While previous reports have shown the enhanced activation of EMT in the worse outcome basal‐like PDAC subtype (Chan‐Seng‐Yue *et al*, 2020), recent reports suggest that the most aggressive tumor cell phenotype is actually conferred by a hybrid EMT state (Pastushenko *et al*, 2021). RNA velocity analyses by Yamazaki *et al* pointed to partial EMT cells as the origin of diverse tumor cell subclusters suggesting that these cells could function as TICs in PDAC and play an essential role in the development of intratumoral heterogeneity. Partial EMT cells were marked by the expression of tyrosine kinase‐like orphan receptor 1 (ROR1) and higher ROR1 levels associated with worse overall survival in TCGA PDAC cohort. Yamazaki *et al* (2023) found that higher levels of ROR1 expression in PDAC TCGA samples correlated with worse overall survival. Functionally, the isolation of tumor cells from xenograft tumors based on ROR1 expression followed by limiting dilution experiments demonstrated that ROR1^high^ cells possessed higher *in vitro* and *in vivo* tumor initiating capacity compared with ROR1^low^ and that they were able to recapitulate the parental tumor heterogeneity. Interestingly, the downregulation of ROR1 resulted in reduced tumor initiation indicating that ROR1 is not only a marker of TICs, but it also plays a functional role in the tumor initiating capacity of PDAC cells. One of the characteristics of TICs is their higher resistance to treatment and enhanced metastatic potential. In line, ROR1^high^ cells were enriched after gemcitabine treatment *in vivo* and ROR1 expression conferred cells the ability to metastasize to lung and mesenteric lymph nodes. Remarkably, the downregulation of ROR1 in combination with gemcitabine treatment resulted in an almost complete tumor regression in preclinical *in vivo* models (Fig 1, left).

**Figure 1:**
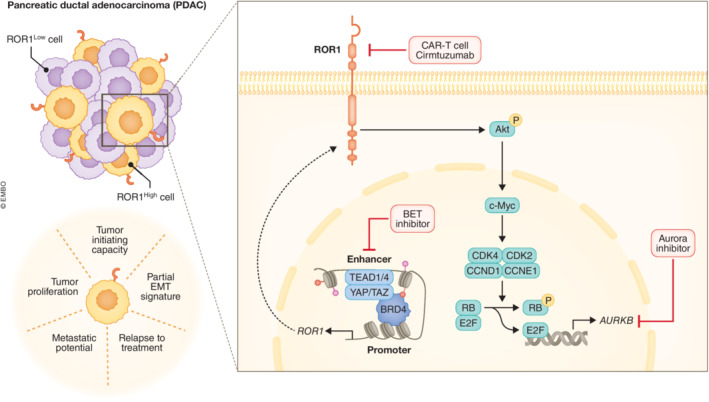
ROR1 expression identifies PDAC TICs and mediates PDAC aggressive phenotype Left: ROR1 expression contributes to PDAC intratumor heterogeneity and malignancy. ROR1 high cells present tumor initiating capacities, partial EMT signature, and are highly proliferative with metastatic potential and resistance to treatment. Right: ROR1 activation is transcriptionally dependent on YAP/BRD4 binding to a novel enhancer region. Once ROR1 is active, it induces Aurora kinase B (AURKB) expression by enabling E2F transcriptional activity via c‐Myc induction. This mechanism has potential therapeutic targetability for the more aggressive PDAC subtype.

To explore the underlying molecular mechanism of ROR1 in driving the TIC phenotype of PDAC cells, Yamazaki *et al* (2023) performed a series of computational analyses together with gain‐ and loss‐of‐function *in vitro* experiments. ROR1 presence increased AKT/MYC signaling to promote E2F‐driven transcriptional activation of Aurora kinase B (AURKB). AURKB appeared to be essential in the ROR1 TIC‐promoting role as pharmacological inhibition of AURKB dramatically reduced the capacity of ROR1^high^ cells to initiate organoid formation. Based on H3K4me1 and H3K27ac histone modifications and transposase‐accessible chromatin organization (ATAC‐Seq), the authors identified a novel enhancer region upstream of the ROR1 promoter. Reporter assays demonstrated that this new enhancer region regulates ROR1 expression and that chromatin states of the enhancer correlate with the ROR1 status of different tumor cell subpopulations. Aiming to uncover which factor could be binding to the novel enhancer region to drive ROR1 expression, Yamazaki *et al* (2023) analyzed the computational overlap of H3K4me1 and H3K27ac distribution between S2‐VP10 PDAC genome and the ChIP‐Atlas database. Together with experimental analyses, the authors identified direct binding of the transcription factor YAP and the BET family member BRD4 to ROR1 enhancer and demonstrated that both factors interact with each other. Collectively, these results provide evidence of a YAP/BRD4 axis triggering ROR1 expression in a subset of PDAC cells. In turn, ROR1 expression activates AKT/MYC/E2F/AURKB signaling providing these cells with tumor initiating capacities (Fig 1, right).

The findings from Yamazaki *et al* (2023) open up new perspectives on PDAC patient stratification and treatment. According to the data, ROR1‐expressing TICs could be targeted by AURKB or BET inhibitors. AURKB and BET inhibitors have entered clinical trials encouraged by promising preclinical results. Yet, limited efficacy and strong off‐target effects have precluded their general use (www.clinicaltrials.gov). While stratification of patients according to ROR1 could help overcome these issues, the use of ROR1 inhibitors here emerges as an exciting alternative treatment strategy. Different ROR1‐targeted approaches, such as treatment with the specific antibody cirmtuzumab (Choi *et al*, 2018) or ROR1‐driven CAR‐T therapy (Srivastava *et al*, 2021), have been explored in other malignancies. In this work, the authors reported an increase in ROR1^high^ population upon treatment with gemcitabine, and treatment of gemcitabine in combination with genetic downregulation of ROR1 expression prevented tumor relapse and metastasis. These results suggest an opportunity for combined treatment of gemcitabine and ROR1 blockers in a subset of patients presenting a partial EMT signature. The relation of ROR1 with EMT and malignancy further suggests that ROR1 could be included as a molecular marker of the basal‐like PDAC subtype. Previous reports have indeed found that this subtype associates with epigenetic alterations in genes related to EMT, YAP, MYC, and E2F7 (Lomberk *et al*, 2018) and that BRD4 drives this phenotype in PDAC tumor cells (Tu *et al*, 2021). The work from Yamazaki *et al* (2023) points to a new potential avenue to identify and target the more aggressive cancer cells within the tumor and to treat the most threatening PDAC subtype. Finally, the presence of ROR1 in other tumor types suggests that these findings could potentially be extended beyond PDAC.
